# Wearable Peripheral Electrical Stimulation Devices for the Reduction of Essential Tremor: A Review

**DOI:** 10.1109/access.2021.3084819

**Published:** 2021-05-28

**Authors:** ALEXANDRA KARAMESINIS, ROY V. SILLITOE, ABBAS Z. KOUZANI

**Affiliations:** 1 School of Engineering, Deakin University, Geelong, VIC 3216, Australia; 2 Department of Pathology and Immunology, Department of Neuroscience, Baylor College of Medicine, Houston, TX 77030, USA; 3 Jan and Dan Duncan Neurological Research Institute, Texas Children’s Hospital, Houston, TX 77030, USA

**Keywords:** Essential tremor, treatment, wearable device, sensor, electrical stimulation, algorithm, open-loop, closed-loop

## Abstract

Essential tremor is the most common pathological tremor, with a prevalence of 6.3% in people over 65 years of age. This disorder interferes with a patient’s ability to carry out activities of daily living independently, and treatment with medical and surgical interventions is often insufficient or contraindicated. Mechanical orthoses have not been widely adopted by patients due to discomfort and lack of discretion. Over the past 30 years, peripheral electrical stimulation has been investigated as a possible treatment for patients who have not found other treatment options to be satisfactory, with wearable devices revolutionizing this emerging approach in recent years. In this paper, an overview of essential tremor and its current medical and surgical treatment options are presented. Following this, tremor detection, measurement and characterization methods are explored with a focus on the measurement options that can be incorporated into wearable devices. Then, novel interventions for essential tremor are described, with a detailed review of open and closed-loop peripheral electrical stimulation methods. Finally, discussion of the need for wearable closed-loop peripheral electrical stimulation devices for essential tremor, approaches in their implementation, and gaps in the literature for further research are presented.

## I. INTRODUCTION

Tremor is an involuntary, rhythmic and oscillatory movement of any body part, which may be pathological or physiological [1]. A common pathological tremor is essential tremor, a neurological disorder which has an estimated prevalence of 0.4%, which increases to 6.3% in those aged older than 65 years [2]. A recent consensus statement in 2018 has updated the definition of essential tremor as an isolated tremor syndrome of bilateral upper limb action tremor that has been present for at least three years’ duration. It may also involve tremor in other locations such as the head or voice and is differentiated from other tremor syndromes, including essential tremor plus, by the absence of neurological signs [1]. Both disease-specific and generic quality of life questionnaires have demonstrated that those living with essential tremor have a lower quality of life than those without the disorder, which is worse with greater tremor severity [3], [4]. Essential tremor is an action tremor, meaning that it occurs during movement or when holding a posture against gravity; thus, patients may experience difficulties with eating, writing, and drinking. When comparing tremor-related quality of life between patients with essential tremor and those with Parkinson’s disease, in which tremor more commonly manifests at rest, these tasks were significantly more affected in those with essential tremor [5]. The psychosocial impacts of the disorder must also be considered; many patients with essential tremor experience embarrassment, difficulties at work and symptoms of anxiety and depression [5]. These negative impacts of essential tremor are particularly significant given the prevalence of essential tremor that is refractory to medical treatment and common issues with decreased treatment effect over time and progression of the severity of tremor with advancing age [6].

The diagnosis of essential tremor is usually made based on clinical symptoms and signs with occasional use of qualitative tests such as spiral drawing inspection. Digital systems, such as iPads, have been used to record spiral drawings for analysis and for the potential use of algorithms to increase diagnostic confidence [7], [8]. Emerging quantitative measurement systems including accelerometry, electromyography, and sensors and algorithms that synthesize these measurements are being developed and validated [9]–[11]. Clinical rating scales and questionnaires tend to be used to evaluate essential tremor in the context of research, for example using the TETRAS scale to assess tremor severity, the QUEST scale to assess tremor-related quality of life or the Bain and Findley Tremor ADL scale to assess the impact of tremor on activities of daily living. However, a MDS task force has recommended only one scale as a screening tool, the WHIGET Tremor Rating Scale, version 1, for the detection of abnormal tremor in clinical practice [12].

Current treatments for essential tremor consist of pharmacological treatments, most commonly propranolol and primidone [13]. Almost one third of essential tremor patients who are prescribed pharmacological treatment stopped taking their medication and this figure was similar even in those with severe tremor; this emphasizes the limitations of the available pharmacological treatments [14]. Older essential tremor patients are less able to tolerate these potential side effects of medical treatment, thus narrowing the treatment options in the demographic where the condition is most prevalent [15]. For medically refractory essential tremor, surgical treatments such as deep brain stimulation or thalamotomy may be suitable [13]. Surgical therapies may not be suitable for elderly patients with comorbidities and evolving technologies including biomechanical loading and non-invasive stimulation of peripheral nerves may improve the suppression of tremor symptoms in these patients [16]. Peripheral neuromodulation devices have been shown to reduce tremor power and frequency in preliminary trials, with one study finding that a closed-loop system is more effective at reducing tremor frequency [17], [18]. Only 11.8% of 1418 respondents to a survey posted in the e-newsletter of the International Essential Tremor Foundation in 2015 were satisfied with their care, demonstrating a significant need for improved therapeutic options in essential tremor [19].

To implement a wearable closed-loop system for essential tremor suppression, an accurate sensing mechanism must be used to detect and quantify the tremor and a signal, whether through biomechanical loading or electrical stimulation, must be applied in response using an appropriate control algorithm. This paper provides an overview of essential tremor, current approaches to measuring the key symptoms of essential tremor, and an analysis of wearable peripheral electrical stimulation devices used to regulate this condition. This review adds to the literature by comparing peripheral electrical stimulation approaches and their currently limited implementation in wearable devices, and providing suggestions based on this synthesis of the direction of future development of wearable peripheral electrical stimulation devices for essential tremor. Included references were found by searching PubMed, Google Scholar and IEEE Xplore using terms including: essential tremor, stimulation, open-loop, closed-loop, neuromodulation, wearable, detection, management, reduction and suppression. Nine studies investigating the use of peripheral electrical stimulation for essential tremor suppression were analyzed; studies which did not include human trials of the intervention on essential tremor patients were excluded. From these studies, two wearable devices were identified and compared.

## II. PATHOPHYSIOLOGY OF ESSENTIAL TREMOR

The pathophysiology of essential tremor is not fully understood and this may contribute to the lack of reliable treatment options for the condition. In recent years, studies have demonstrated that various structural changes in the brain may be linked to essential tremor, particularly in the cerebellum. However, no clear patterns of structural changes have been consistently identified across neuroimaging studies [27]. The heterogeneity of these structural changes in essential tremor is reflected by the heterogeneity in studies on the genetic basis of the condition; although a family history is a demonstrated risk factor for the condition, no single susceptibility gene has been consistently identified [28]. It is now well-established that functional changes in the cerebello-thalamo-cortical network may be central to the generation of essential tremor, with oscillations in this area hypothesized to arise from a dysfunction in the motor controller, hence leading to action tremor [29]. A possible explanation for this phenomenon is that GABA, an inhibitory neurotransmitter, has lower levels in the cerebellum in essential tremor patients as identified in multiple studies, thus allowing excessive neurological activity [30]. Accordingly, recent work in mice demonstrated that altering Purkinje cell to cerebellar nuclei activity resulted in tremor, and that therapeutically interrupting this abnormal oscillatory neural activity eliminated tremor [31]. Pedrosa *et al*. recently mapped the afferent and efferent contributions to essential tremor in the ventral thalamus using DBS micro-electrode recordings simultaneously with sEMG recordings of the forearm muscle. Afference was determined by the ability of the signal to be predicted by the peripheral tremor, as opposed to efference which described signals that were predominantly predictive of the peripheral tremor. Their findings suggest that a phase difference between afferent and efferent signals may result in an increased tremor amplitude [32]. Thus, modulating the afferent input using sensory-level peripheral electrical stimulation devices presents a promising, and potentially more comfortable, method of essential tremor treatment, as opposed to modulating the muscle contractions to cancel out the tremor with a higher stimulation amplitude, which is the approach of functional electrical stimulation systems.

The central oscillations generated in the cerebello-thalamo-cortical network propagate to spinal motor neurons which stimulate antagonist muscle groups in the arm and forearm resulting in pathological upper limb tremor with a frequency in the middle and high ranges compared to other tremors, often listed as 4-12 Hz or 3-10 Hz [33], [34]. Puttaraksa *et al.* have demonstrated that the phase difference in inputs to antagonist muscle groups causing tremor activation vary over a short timeframe, lending weight to the use of a closed-loop real-time system for out-of-phase electrical stimulations [33]. Mathematical models are being used to further investigate the pathophysiology of essential tremor, for example, to consider the impact of tremulous activity in different muscle groups on the tremor exhibited at each degree of freedom in the upper limb and to investigate the possibility of a combination of central and peripheral neurological factors on the generation of tremor [35], [36]. Pigg *et al*. used motion capture sensors to characterize tremor in the 7 main degrees of freedom in the upper limb of 22 essential tremor patients, finding that the degrees of freedom with the greatest amount of tremor are wrist flexion/extension and forearm pronation/supination. A secondary finding of the study is that no difference in the frequency of tremor was observed between different degrees of freedom in each subject [37].

## III. ARCHITECTURE OF WEARABLE PERIPHERAL NERVE STIMULATION DEVICES

**FIGURE 1.**
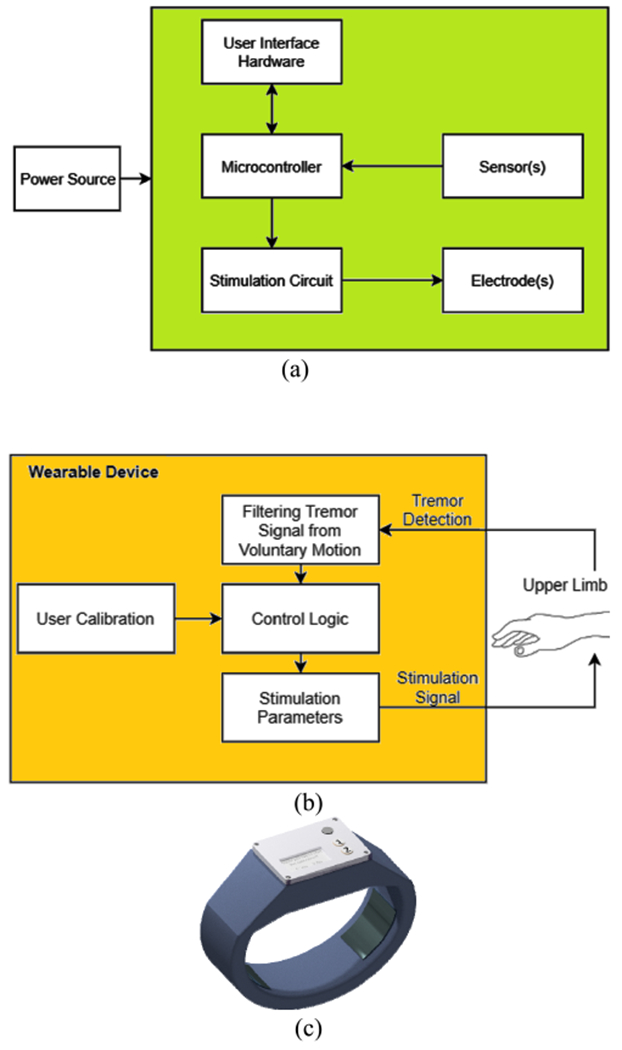
(a) Hardware architecture of wearable devices for essential tremor suppression. (b) Functional block diagram of wearable devices for essential tremor suppression. (c) A prototype wrist-worn device for essential tremor suppression, designed by the authors.

In order for a wearable device to deliver non-invasive peripheral nerve stimulation for essential tremor in a closed-loop configuration, it must include the following hardware subsystems: tremor sensor, on-board processor, stimulation circuit and electrodes, rechargeable power supply, and user interface such as buttons and a display,. The software must be able to record and process the tremor measurements and apply an appropriate stimulation signal based on the control approach. The initial user calibration, such as setting the maximum pulse amplitude to be applied, should also be considered in the control approach. A generic architecture for wearable devices for essential tremor suppression is presented in Figure 1(a–b). A typical wearable device for essential tremor suppression is shown in Figure 1(c).

## IV. MEASUREMENT OF TREMOR

### A. SURFACE ELECTROMYOGRAPHY

Surface electromyography (sEMG) involves measuring the electrical excitation of underlying muscles using electrodes placed on the skin. Depending on the electrodes used, skin preparation and disposal of electrodes before and after each use may be required [44]. The signal obtained during tremorous motion may be visualized as bursts of electrical activity in the muscles, often alternating between two opposing muscle groups [44]. In essential tremor, these bursts have a duration of approximately 50 to 200 milliseconds, whilst in Parkinson’s disease the duration range is smaller, between 50 and 150 milliseconds; due to these feature differences, sEMG has been extensively researched as a tool for distinguishing tremor syndromes from one another, however currently there is no validated method used in clinical practice [44]. Basu *et al*. have demonstrated that sEMG recording of the forearm muscles may be used to predict tremor onset in essential tremor patients prior to the patient perceiving the tremor [45]. Whilst suggested for implementation for closed-loop deep brain stimulation, this entropy-based non-invasive measurement and processing system could be used to create a non-invasive system for the attenuation of tremor when combined with peripheral non-invasive neuromodulation. More recently, Basu *et al*. have combined the use of sEMG with accelerometer data to increase essential tremor prediction accuracy to 85.7% [46].

The integration of surface electromyography into wearable tremor suppression devices proves challenging for two key reasons: the placement of electrodes needs to be specific to each patient and there is interference between peripheral nerve stimulation signals and surface EMG readings [47]. Zhang *et al*. suggest three stages of processing for raw sEMG data to filter noise, extract the tremor pattern from voluntary movement and finally to create a smooth EMG envelope signal [48]. If tremor characteristics such as frequency and amplitude need to be determined based on the sEMG signal, processing such as the use of the iterated Hilbert transform as described by Dideriksen *et al*. and implemented by Gallego *et al*. have been attempted [49], [50].

### B. WEARABLE MOTION TRANSDUCERS

Accelerometers and gyroscopes are commonly used to measure tremor, given their prevalence in smart devices such as phones and watches and availability as compact micro-electromechanical packages [51]. These sensors enable the amplitude and frequency of the tremor in each axis to be calculated from the measurements, after signal processing occurs to remove the superimposed voluntary motion signal. Although accelerometers have historically been used more commonly for this purpose, a key issue is the presence of gravitational artefacts when components of the motion are rotational, which is the case in tremor, where the motion occurs around a joint. In order to address this issue, multiple accelerometers may be mounted in strategic locations on a body part and the data from each synthesized to remove the gravitational artefact; however, this may not be convenient for long-term in home monitoring [52]. Two wearable devices using peripheral nerve stimulation for tremor suppression use 3-axis accelerometers located on the forearm near the wrist to measure the patients’ tremor [18], [53].

When using gyroscopes to measure tremor, two gyroscopes are often used, placed on the dorsum of the hand and the dorsum of the forearm near the wrist; this allows for direct measurement of the angular velocity of flexion and extension at the wrist, although may prove cumbersome to implement in a wearable device [47], [54]. Two identified studies have used clinical trials to determine the accuracy of inertial sensors in measuring tremor in comparison to clinician-rated scales; both studies showed some statistically significant positive correlations between the measured severity and rated severity, however, neither had the same findings for every task performed by participants [55], [56]. One study by McGurrin *et al*. combining both gyroscope and accelerometer measurements from the dorsum of the hand demonstrated that the measurement of tremor using these sensors correlated moderately to strongly with clinician-rated TETRAS scores; the findings were statistically significant for tasks such as spiral drawing, hand-writing and dot approximation [55]. However, the algorithm used to determine the tremor frequency and amplitude operated in two distinct stages, including initially determining the typical frequency range for each participant, which is not suitable for implementation in a real-time closed-loop system [55]. The study conducted brief testing using a robotic arm to determine the accuracy of the amplitude estimated from the accelerometer and gyroscope measurements, and found errors of +−0.02cm and +− 0.05 degrees respectively [55]. The NetMD study used sensors in a commercially available smart watch, comparing the measured tremor severity to the clinician-rated FTM-TRS scale, and found moderate to strong statistically significant correlation in resting, postural and water-pouring tasks, with no correlation found in a finger-to-nose maneuver task [56].

Devices that implement accelerometry or gyroscope sensors for the detection of tremor utilize high-pass filtering to obtain the tremor signal and differentiate it from voluntary movements, which tend to have a much lower frequency than the tremor being measured [18], [51]. Other techniques that may be considered for signal processing and removal of voluntary movement measurements using inertial sensors include the adaptive band-pass filter proposed by Popovic *et al*. and the more common weighted frequency Fourier Linear Combiner [57], [58]. In a systematic review of four different groups of signal processing approaches for MEMS gyroscopes, adaptive-based filters such as those mentioned previously were found to be most suitable for tremor modeling and estimation; these may be implemented in real time using only a MEMS gyroscope, or with additional measurements from an accelerometer [58]. Temperature bias may impact measurements using gyroscopes, but this may be addressed by using gyroscopes with an internal compensator for temperature [44].

### C. OTHER METHODS

Digitizing tablets may be used to assess tremor through the recordings of handwriting or spiral drawings; the translational displacement of a pen on the surface of the tablet over time may be used to determine the frequency and amplitude of the tremor [51]. These systems, whilst useful for intermittent monitoring of tremor severity and progression, are unable to be used for continuous quantification of essential tremor due to the practical issue of requiring a patient to draw on a tablet to obtain a sample.

Optical sensors for tremor detection include videos and optoelectronic devices [9], [44]. A commercially available device, Leap Motion, uses two infrared sensors to detect motion and was validated for finger tremor characterization in four patients with essential tremor, providing an option to objectively determine tremor amplitude in a clinical setting [9]. However, optical sensors are not suitable for implementation for tremor detection as a wearable device; wearable markers may be used in conjunction with an external camera or optical sensor [44].

Force transducers could also be used for tremor detection; however, these sensors are more difficult to implement than those mentioned previously and are more expensive than motion sensors [44].

Electroencephalography (EEG) signals may be used to identify the intention for voluntary movement, in order to assist in separating tremor signals from voluntary movement detected by another of the previously mentioned sensors [50]. The use of EEG in a wearable device for tremor suppression has been implemented by Gallego *et al*., along with sEMG and IMU to characterize the tremor [50].

## V. NOVEL INTERVENTIONS FOR ESSENTIAL TREMOR

### A. BRIEF OVERVIEW OF MECHANICAL INTERVENTIONS

Biomechanical loading devices and other orthoses have been developed to reduce tremor symptoms. These devices have been found to be heavy and cumbersome, making the patient less likely to engage with this treatment option than the previously mentioned alternatives [59]. Devices such as tremor cancellation spoons have been shown to be effective; however they require that the patient has a different device for each specific task, such as eating, writing or using a tablet device, thus increasing cost and effort, and decreasing practicality [60].

### B. BRIEF OVERVIEW OF NON-INVASIVE CENTRAL STIMULATION

Various types of non-invasive central stimulation may provide further treatment options for essential tremor. Trans cranial magnetic stimulation of the cerebellum has been studied in a small number of patients, and demonstrates acute motor improvement in essential tremor; however, not all studies have shown significant improvements compared to sham stimulation and whether long-lasting improvements can occur is uncertain [61], [62]. Another form of central stimulation, trans cranial direct current stimulation has shown a significant improvement in TETRAS scores in 6 essential tremor patients after a 15 session treatment course spanning across a 50 day period [63]. Given that studies into this area have not been large, further research into the various stimulation methods and parameters is required, including whether such methods could be implemented into wearable or at-home devices to improve patient engagement [64].

### C. PERIPHERAL ELECTRICAL STIMULATION

Peripheral electrical stimulation, either functional or below the motor threshold, has been studied as a treatment of essential tremor since the early 1990s, when Prochazka *et al*. created a closed-loop functional electrical stimulation system which attenuated essential tremor by an average of 73% in three patients [65], [66]. In 1993, Britton *et al*. demonstrated that essential tremor, tremor associated with Parkinson’s disease and mimicked tremor could be modulated with transcutaneous supramaximal stimulation of the median nerve close to the elbow [67]. A consistent finding across a number of studies is that patients with a more severe tremor initially, for example as rated by a clinician on the TETRAS scale, experience a greater improvement in their function with the use of peripheral electrical stimulation than those patients with a lower severity score initially [17], [18], [53]. Although peripheral electrical stimulation with intramuscular electrodes may decrease muscle fatigue and have a higher stimulation pain threshold, intramuscular electrodes are invasive and thus difficult to implement in a wearable device, and therefore are not discussed in detail in this review [65].

Table 1 compares approaches to peripheral nerve stimulation in a number of studies and their effectiveness at suppressing tremor, which ranged from 42-81%, with most studies showing that many patients showed at least some improvement [17], [18], [47], [53], [68], [69]. One study implies the longevity of tremor suppression effects by using 40 minutes of stimulation twice per day over three months; there was a statistically significant improvement between the pre-session scores from the first visit and the third visit [53]. In Table 1, ‘wearable hardware’ is defined as a single device including the necessary sensors and electrodes to conduct the peripheral nerve stimulation; it may include onboard processing or communicate via wireless communications with a computer or other device for processing. It does not include devices that are required to be physically attached to computers, EMG amplifiers or other hardware. The various approaches detailed in Table 1 include open-loop stimulation, stimulation modulated by tremor frequency recorded during a calibration window and closed-loop ON/OFF control of the stimulation based on the presence of tremor at a given time [17], [18], [47], [53], [68], [69]. However, there is an absence in the literature of a wearable closed-loop system that takes into account real-time changes in factors such as tremor frequency, amplitude and presence of tremor and changes the stimulation signal characteristics in response.

One benefit of closed-loop approaches to electrical stimulation for tremor suppression is the lower muscle fatigue compared to continuous stimulation [65]. However, a challenge in implementing continuous closed-loop control is that the tremor will manifest differently once the stimulation is applied and this will interfere with developing an appropriate signal, hence the use of a 1 second recording window and 3 second stimulation window in some studies [47], [68]. Closed-loop control strategies that have been applied and tested using software simulation or patients with other types of tremor include fuzzy logic control, repetitive control and neural oscillator-based control, which achieved simulated tremor suppression of about 85%, 80.7-88.4% in a patient with MS, and 90%, respectively [48], [70], [71].

Table 2 lists the stimulation parameters used in a number of non-invasive peripheral stimulation trials including essential tremor patients. Only one study has compared the effectiveness of different stimulation parameters in the same patient group, with a small study size (n = 9) limiting the significance of the findings that 50 and 100 Hz stimulation frequencies and 25% and 37.5% duty cycles for closed-loop stimulation reduced tremor frequency, and 100 Hz and 37.5% duty cycle for open-loop was best at reducing tremor power [18]. The most common approach to tremor suppression with electrical stimulation is to stimulate the muscles or nerves out-of-phase to the relevant muscle activation, to create a noise-cancelling effect on the tremor [47], [68], [69]. Similarities in the values of each stimulation parameter listed in Table 2 exist in the studies, with the exception of the pulse rate, with values between 25 Hz and 200 Hz used in the studies. However, the justification for each parameter choice is not available.

Non-invasive peripheral electrical stimulation is also used in rehabilitation robotics to restore motor capabilities; analysis of the approaches and circuitry in these areas may provide applicable methodologies to wearable tremor suppression devices. For example, the Hyper project has developed an optimized circuit using a voltage to current transconductance amplifier for generating a balanced biphasic rectangular waveform, which has been shown to lead to less muscle fatigue compared to other waveform shapes [72].

Adverse effects of peripheral electrical stimulation includes changes in sensation such as tingling, stinging or pain distal to the area where the stimulation was applied and redness, itchiness or swelling at the location of the electrodes [17], [18], [53], [65]. In terms of potential dangers of using this type of device at home, one study reported a fall that “may have been related to the device”, however the context of this event was not described [53]. There is a general caution about applying external electrical stimulation to patients with implanted pacemaker devices and implantable cardioverter defibrillators (ICDs) [73]–[75]; a recent systematic review demonstrated that use of electrical stimulation on the upper body and upper limb is significantly more likely to result in incorrect sensing and/or shock by ICDs and pacemakers than electrical stimulation on the lower limb [74]. Another contributing factor to a higher likelihood of incorrect shock demonstrated by an experimental study is bilateral use of electrical stimulation as opposed to unilateral use [75].

In addition to the potential for adverse effects, there are limitations of the included studies and experimental devices. The electrode positions in most of the devices need to be calibrated for each patient, often involving using a stimulation probe to determine the correct position; this creates a challenge in making a wearable device that is easy for a patient to take on and off as needed [65], [76]. However, the Cala device tested by Isaacson *et al*. and Pahwa *et al*. approaches this issue by using three different standard sizing options and reusable electrodes that are built into the device strap [17], [53]. In order to apply electrical stimulation to multiple pairs of agonist and antagonist muscles and have the benefit of peripheral electrical stimulation at multiple joints, increasing amounts of hardware are required, reducing the likelihood of having a comfortable wearable device [65], [66]. In parallel to the issue of electrode placement is the difficulty in sensor placement for accurate displacement measurement each time a sensor is worn by the patient [65]. This issue could be circumvented by using an algorithm for determining sensor location in real-time without context as developed by Lambrecht *et al*. [77]. Another limitation in the reviewed devices is that the initial calibration periods had to be carried out manually to determine a comfortable stimulation threshold for each individual patient [65].

Some studies found that one or more of the essential tremor patients failed to respond at all to the electrical stimulation, demonstrating that this treatment, similarly to other treatments for essential tremor, may not be effective for all patients [47], [68]. The study by Kim *et al*. tested only one type of movement, a bean-transfer task, limiting the implications of their findings [18]. A significant hindrance to the quality of the included studies is the lack of controlled trials, except for the study by Pahwa *et al*. [17]. The majority of the studies listed in Table 1 have a small sample size, except for the studies of the Cala device conducted by Pahwa *et al*. and Isaacson *et al*.; the tremor suppression results show some motor improvement in tremor in all of the included studies, indicating that there is clinical potential for this treatment [17], [53].

## VI. DISCUSSION

Novel treatment approaches for essential tremor patients have the potential to benefit a large number of patients who cannot be adequately treated with medication and who may not be suitable or may not wish to undergo significant surgical intervention. As demonstrated by the resistance from patients to use external mechanical loading devices and related assistive technologies, more discreet and comfortable solutions need to be developed; hence, the recent push for the development of wearable electrical stimulation technologies for tremor suppression. Table 3 compares the characteristics of the two wearable devices identified in the literature.

**FIGURE 2.**
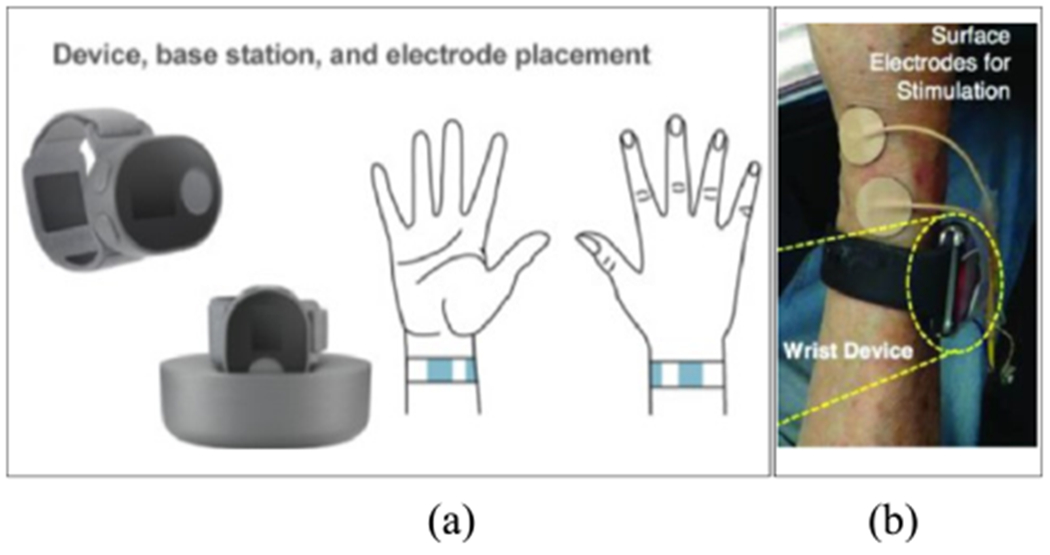
(a) A wearable device with a charging station and electrodes in the band [53]. (b) A wearable device that has external electrodes and a small form factor [18].

Figure 2 shows the two wearable devices, with the key differences being the electrode placement; one device offers electrodes built-in to a wristband which needs to be replaced every three months [53], and the second device has electrodes that need to be placed on the skin manually during calibration [18]. Both of these devices require study personnel to assist with the setup and calibration of the devices, creating a barrier to patient use. Furthermore, one of the devices uses a computer to process the sensor data and determine the stimulation signal, which will limit the number of patients who could use the device based on whether they have access to a computer and appropriate software. The battery life of the device by Kim *et al*. is sufficient for a patient to wear the device throughout the day and charge it overnight, which is a strength of the system; battery life in peripheral electrical stimulation devices can be further improved with the use of closed-loop control, as tremor is not continuously present throughout the day in all patients.

When comparing sensors for tremor measurement that may be implemented in wearable devices, the practicality, accuracy, reliability and sampling rates of the options must be considered. A particular limitation in this area is that, whilst sEMG can provide very useful measurements which allow tremor prediction, rather than only detection, applying electrical stimulation to the same muscles can result in interference in the measured signals. In addition to this, sEMG requires further electrodes to be securely placed on the patient, increasing the size of a potential wearable device and decreasing the ease of use for the patient. The ability to extract the frequency and amplitude of tremor from inertial measurement units, combined with their low cost and small size make them a logical choice for tremor detection. Theoretically, the placement of these sensors should allow for measurement of the tremor at the joint moved by the muscles or equivalent nerves which the stimulation is applied to; however, in the two identified wearable devices for sensory stimulation, a single sensor was used to determine the tremor characteristics at the forearm only, whereas the nerves being modulated would move the wrist. Although the reasoning is not detailed in the publications by Kim *et al*. and Isaacson *et al.,* this sensor placement may be justified by the finding that tremor frequency is similar in all joints of the upper limb in an individual patient [18], [37], [53].

Reflecting on the data in Table 1, it is evident that the more recent approaches using sensory level stimulation have not produced as high a level of tremor suppression as the functional electrical stimulation approaches. However, these sensory stimulation studies have used control approaches mimicking that of the out-of-phase muscle stimulation, despite being based on a different pathophysiological theory. Given that some tremor suppression was still achieved in the majority of patients, this would suggest that sensory stimulation approaches are underperforming because they have not been fully investigated as a separate entity and could have greater tremor suppression if optimized, and could be widely adopted by patients as a more comfortable alternative to functional electrical stimulation. Due to the largely unknown nature of the pathophysiology of essential tremor, and the variation in the patient responses to peripheral electrical stimulation both within and across the studies identified in this work, an adaptive control approach which seeks the ideal stimulation approach for each individual patient over time would be a logical next step to take in this field.

## VII. CONCLUSION

There is significant potential for non-invasive peripheral electrical stimulation to reduce tremor amplitude in essential tremor patients, particularly to improve symptoms in the 88.2% of essential tremor patients who are not satisfied with their treatment. However, this treatment is most effective at the time of, or soon after, stimulation is applied and thus must be integrated into a patients’ daily life to be effective. This review demonstrates the need for and the potential development of approaches for an upper limb essential tremor suppression device that is comfortable and easy to use and employs continuous closed-loop control strategies to maximize tremor suppression whilst minimizing muscle fatigue for the patient. As demonstrated by this review, all aspects required to implement such a system, including sensor types and processing, stimulation signal generation and closed-loop control strategies, have been studied as individual subsystems, or as part of non-wearable devices, with promising results albeit small sample sizes. To improve on current approaches in a way that will be practical and comfortable for patient implementation, adaptive control approaches for sensory-level stimulation should be developed and tested in larger patient populations.

## Figures and Tables

**TABLE 1.** Human experimental studies of tremor suppression using non-invasive peripheral stimulation.

Study	n (Number of Patients)	Wearable/Non-wearable Hardware	Electrodes (Number Excluding Negative Electrode, Placement, Type)	Sensors (Number, Type, Sampling Rate)	Tremor Suppression	Comments
**Sensory Stimulation**						
S. Dosen et al., 2015 [68]	6 (4 PD, 2 ET)	Non-wearable	2, point with the strongest motor response from a probing electrode; for wrist extensors this was close to lateral epicondyle and for wrist flexors, proximal to the elbow crease and medially, PALS Platinum electrodes	2, sEMG, 1kHz	35-48% (sensory stimulation in 5 patients) 46-81% (motor stimulation in 5 patients)	1 ET patient failed to have any tremor suppression. Approach: 3 second stimulation window, 1 second recording window. Iterative Hilbert Transform used for tremor demodulation.
Pahwa et al., 2019 [17]	77 ET patients (40 received treatment stimulation, 37 received sham stimulation)	Wearable	2, overlying the path of the median and radial nerves close to the wrist, 2.2 cm × 2.2 cm square hydrogel electrodes	N/A, accelerometers, N/A	46% reduction in tremor amplitude in the treatment group compared to 24% reduction in tremor amplitude in the sham group.	Approach: 40 minutes of stimulation after a short calibration to the patient’s dominant tremor frequency with tasks and tremor ratings completed immediately following this.
Isaacson et al., 2020 [53]	263 ET patients enrolled, 205 patients completed at least one stimulation session per day on 78% of the days on average.	Wearable	2, overlying the path of the median and radial nerves close to the wrist, 2.2 cm × 2.2 cm square hydrogel electrodes	N/A, accelerometers, N/A	92% of patients had an improvement ratio greater than 1 from pre- to post-session.	Approach: 40 mins stimulation twice per day over 3 months.
Kim et al., 2020 [18]	9 ET patients (1 participated in 2 separate sessions)	Wearable	1, overlying the site of the radial nerve close to the wrist, 0.8” round transcutaneous electrical nerve stimulation unit electrodes (Syrtenty)	1, 3-axis Accelerometer (LSM303D), 100 Hz	42.17% +− 3.09% reduction in tremor power.	Approach: For the closed-loop trials, active tremor periods and tremor phase were analysed in real-time to determine when to apply the nerve stimulation.
**Motor Stimulation**						
Javidan et al., 1992[66] and Prochazka et al., 1992 [65]	24 (3 ET, 4 PD and 6 other)	Non-wearable	2, overlying biceps muscle and overlying triceps muscle, 2 cm × 3cm pre-gelled self-adhesive electrodes (Chattanooga Corp.)	1, low-noise optical displacement transducer monitoring a pivoting armrest or strain gauge, N/A	73% tremor suppression in ET patients.	Approach: Manual calibration to ensure equal flexor and extensor offset torques was done initially for each subject. A filter designed to maximize open-loop gain in the 2-5 Hz frequency range and minimize phase lag in the 0-10 Hz frequency range was used and the stimulation was applied out-of-phase to the tremorogenic muscles.
Gallego et al., 2011[50]	1	Non-wearable	N/A, array of electrodes on forearm and arm, sewn electrodes in e-textile (SMARTEX)	1, 16-channel EEG, N/A 1, 128-channel sEMG, N/A 2, IMU, N/A	60% tremor attenuation	Approach: open-loop stimulation using an electrode array to increase joint impedance.
Widjaja et al., 2011 [69]	1	Non-wearable	2, image shows electrodes on the dorsum of the forearm, N/A	2, sEMG, 1 kHz 1, 3-axis accelerometer, 1 kHz	57% tremor power reduction	Approach: real-time closed-loop ON/OFF control. An algorithm determines first if the sEMG signal is large enough to be considered tremor, then combines accelerometer and sEMG phase estimations to determine when to start the signal to ensure it is out of phase.
Popović Maneski et al., 2011 [47]	7 (some PD, some ET)	Non-wearable	2, “dorsal and volar side of the forearm over the motor points of the wrist extensors and flexors”, PALS round electrodes	2, gyroscope,1kHz	67 +/− 13% (average tremor suppression in 6 patients)	1 patient failed to have any tremor suppression. Approach: 3 second stimulation window, 1 second recording window

Note: N/A = not available, sEMG = surface electromyography.

**TABLE 2.** Stimulation parameters for non-invasive peripheral stimulation.

Study	Constant Current or Constant Voltage Stimulation	Pulse Rate	Pulse Duration	Pulse Amplitude	Channel Delay	Out-of-phase or In-Phase	Duty Cycle
Popović Maneski et al., 2011 [47]	Constant current, (voltage maximum limit: 95V)	40 Hz	250us	Minimal value (between 0 and 50mA) able to induce full flexion or extension at the wrist.	0-1000ms	Out-of-phase	N/A
Dosen et al., 2015 [68]	Constant current. (voltage maximum not reported)	100 Hz	300 us	Current amplitude to obtain flexion or extension of the joint (motor stimulation). Mean (wrist flexor) 15 +− 3 mA, mean (wrist extensor) 18 +− 5 mA. Highest current amplitude which did not cause muscle contraction (sensor stimulation). Mean (wrist flexor) 9 +− 3 mA, mean (wrist extensor) 10 +− 3mA.	N/A	Out-of-phase	N/A
Widjaja et al., 2011 [69]	Constant current. (voltage maximum not reported)	25 Hz	200 us	23 mA	N/A	Out-of-phase – possibly only applied to wrist extensors.	N/A
Pahwa et al., 2019 [17] and Isaacson et al., 2020 [53]	Constant current. (voltage maximum not reported)	150 Hz	300 us biphasic pulses, 50 us interpulse duration	Highest tolerable stimulation level (below threshold for muscle contraction). Mean 5.4mA +− 2.9	N/A	Alternating between stimulating the median and radial nerve at a frequency equal to the patient’s dominant tremor frequency as measured during a calibration period.	N/A
Kim et al., 2020 [18]	Constant voltage. (current limits not reported)	50, 100 or 200 Hz	200 us (biphasic, 100us per phase)	Between 3.57 V to 17.33V depending on the minimal strength of stimulation that could be discerned by the subject.	N/A	In-phase for the closed-loop trials, based on the dominant tremor frequency.	12.5%, 25% and 37.5% of the tremor cycle were trialled.
Prochazka et al., 1992 [65]	Constant current. (voltage maximum not reported)	30 Hz	200 us	Between 20mA to 100mA, amplitude-modulated based on filtered tremor measurement signal.	N/A	Out-of-phase	N/A

Note: N/A = not available

**TABLE 3.** Comparison of wearable device characteristics.

Study	Stimulati on Level	Aesthetic Description	Processing Requirements	Mass	Battery Life	Calibration Requirements	Other Notes
Kim et al., 2020 [18]	Sensory	Wristwatch-style device with disposable electrodes.	Processing completed on external computer, connected via wireless transceiver.	36 g	A 3-hour charge lasts approximately 20 hours.	Initial tests by study personnel to correctly place electrodes and find correct stimulation amplitude.	N/A
Pahwa et al., 2019 [17] and Isaacson et al., 2020 [53]	Sensory	Wristwatch-style device with electrodes built into wristband.	On-board processing whilst stimulation occurs – data for analysis is collected wirelessly from the device whilst charging at a base station.	N/A	A 4 hour charge lasts for 5, 40-minute, stimulation sessions.[78]	Each patient needed to be fitted with a small, medium or large wristband which is specific to R or L hand. Study personnel manually increased stimulation amplitude initially.	The wristband must be replaced every 3 months. The cost of this device is USD$3200+$157 monthly band subscription [78].

Note: N/A = not available

## References

[1] Bhatia KP, Bain P, Bajaj N, Elble RJ, Hallett M, Louis ED, Raethjen J, Stamelou M, Testa CM, and Deuschl G, “Consensus statement on the classification of tremors. From the task force on tremor of the international Parkinson and movement disorder society,” Movement Disorders, vol. 33, no. 1, pp. 75–87, 1. 2018. 29193359 10.1002/mds.27121 PMC6530552

[2] Louis ED and Ferreira JJ, “How common is the most common adult movement disorder? Update on the worldwide prevalence of essential tremor,” Movement Disorders, vol. 25, no. 5, pp. 534–541, 4. 2010. 20175185 10.1002/mds.22838

[3] Chandran V and Pal PK, “Quality of life and its determinants in essential tremor,” Parkinsonism Rel. Disorders, vol. 19, no. 1, pp. 62–65, 1. 2013. 10.1016/j.parkreldis.2012.06.011 22771281

[4] Nguyen HV, Ngian V, Cordato D, Shen Q, and Chan DKY, “Quality of life in a random sample of community dwelling older patients with essential tremor,” Acta Neurol. Scandinavica, vol. 116, no. 5, pp. 289–292, 11. 2007. 10.1111/j.1600-0404.2007.00863.x 17883423

[5] Louis ED and Machado DG, “Tremor-related quality of life: A comparison of essential tremor vs. Parkinson’s disease patients,” Parkinsonism Rel. Disorders, vol. 21, no. 7, pp. 729–735, 7. 2015. 10.1016/j.parkreldis.2015.04.019 PMC4764063 25952960

[6] Shanker V, “Essential tremor: Diagnosis and management,” BMJ, vol. 366, p. l4485, 8. 2019. 31383632 10.1136/bmj.l4485

[7] Sisti JA, Christophe B, Seville AR, Garton ALA, Gupta VP, Bandin AJ, Yu Q, and Pullman SL, “Computerized spiral analysis using the iPad,” J. Neurosci. Methods, vol. 275, pp. 50–54, 1. 2017. 27840146 10.1016/j.jneumeth.2016.11.004 PMC5308231

[8] Lin P-C, Chen K-H, Yang B-S, and Chen Y-J, “A digital assessment system for evaluating kinetic tremor in essential tremor and Parkinson’s disease,” BMC Neurol, vol. 18, no. 1, p. 25, 3. 2018. 29523097 10.1186/s12883-018-1027-2 PMC5845296

[9] Chen K-H, Lin P-C, Chen Y-J, Yang B-S, and Lin C-H, “Development of method for quantifying essential tremor using a small optical device,” J. Neurosci. Methods, vol. 266, pp. 78–83, 6. 2016. 27058772 10.1016/j.jneumeth.2016.03.014

[10] Mykhalyk D, Mudryk I, Hoi A, and Petryk M, “Modern hardware and software solution for identification of abnormal neurological movements of patients with essential tremor,” in Proc. 9th Int. Conf Adv. Comput. Inf. Technol. (ACIT), 6. 2019, pp. 183–186.

[11] Luft F, Sharifi S, Mugge W, Schouten AC, Bour LJ, van Rootselaar A-F, Veltink PH, and Heida T, “A power spectral density-based method to detect tremor and tremor intermittency in movement disorders,” Sensors, vol. 19, no. 19, p. 4301, 10. 2019. 10.3390/s19194301 PMC6806079 31590227

[12] Elble R, Bain P, Forjaz MJ, Haubenberger D, Testa C, Goetz CG, Leentjens AFG, Martinez-Martin P, Traon AP-L, Post B, Sampaio C, Stebbins GT, Weintraub D, and Schrag A, “Task force report: Scales for screening and evaluating tremor: Critique and recommendations,” Movement Disorders, vol. 28, no. 13, pp. 1793–1800, 11. 2013. 24038576 10.1002/mds.25648

[13] Ferreira JJ, Mestre TA, Lyons KE, León JB, Tan E, Abbruzzese G, Hallett M, Haubenberger D, Elble R, and Deuschl G, “MDS evidence-based review of treatments for essential tremor,” Movement Disorders, vol. 34, no. 7, pp. 950–958, 7. 2019. 31046186 10.1002/mds.27700

[14] Louis ED, Rios E, and Henchcliffe C, “How are we doing with the treatment of essential tremor (ET)? Persistence of patients with ET on medication: Data from 528 patients in three settings,” Eur. J. Neurol, vol. 17, no. 6, pp. 882–884, 6. 2010. 20067514 10.1111/j.1468-1331.2009.02926.x PMC2889923

[15] Louis ED, “The roles of age and aging in essential tremor: An epidemiological perspective,” Neuroepidemiology, vol. 52, nos. 1–2, pp. 111–118, 2019. 30625472 10.1159/000492831

[16] Hedera P, “Emerging strategies in the management of essential tremor,” Therapeutic Adv. Neurological Disorders, vol. 10, no. 2, pp. 137–148, 2. 2017. 10.1177/1756285616679123 PMC5367648 28382111

[17] Pahwa R, Dhall R, Ostrem J, Gwinn R, Lyons K, Ro S, Dietiker C, Luthra N, Chidester P, Hamner S, Ross E, and Delp S, “An acute randomized controlled trial of noninvasive peripheral nerve stimulation in essential tremor,” Neuromodulation Technol. Neural Interface, vol. 22, no. 5, pp. 537–545, 7. 2019. 10.1111/ner.12930 PMC6766922 30701655

[18] Kim J, Wichmann T, Inan OT, and Deweerth SP, “A wearable system for attenuating essential tremor based on peripheral nerve stimulation,” IEEE J. Transl. Eng. Health Med, vol. 8, pp. 1–11, 2020. 10.1109/JTEHM.2020.2985058 PMC7313727 32596064

[19] Louis ED, Rohl B, and Rice C, “Defining the treatment gap: What essential tremor patients want that they are not getting,” Tremor Other Hyperkinetic Movements, vol. 5, no. 0, p. 331, 8. 2015. 26317044 10.7916/D87080M9 PMC4548969

[20] Louis ED, Bares M, Benito-Leon J, Fahn S, Frucht SJ, Jankovic J, Ondo WG, Pal PK, and Tan E-K, “Essential tremor-plus: A controversial new concept,” Lancet Neurol, vol. 19, no. 3, pp. 266–270, 3. 2020. 31767343 10.1016/S1474-4422(19)30398-9 PMC10686582

[21] Crawford P and Zimmerman EE, “Tremor: Sorting through the differential diagnosis,” Amer. Family Physician, vol. 97, no. 3, pp. 180–186, 2. 2018. 29431985

[22] Louis ED and Dogu O, “Does age of onset in essential tremor have a bimodal distribution? Data from a tertiary referral setting and a population-based study,” Neuroepidemiology, vol. 29, nos. 3–4, pp. 208–212, 2007. 18043006 10.1159/000111584 PMC2824583

[23] Hopfner F, Ahlf A, Lorenz D, Klebe S, Zeuner KE, Kuhlenbaumer G, and Deuschl G, “Early- and late-onset essential tremor patients represent clinically distinct subgroups,” Movement Disorders, vol. 31, no. 10, pp. 1560–1566, 10. 2016. 27384030 10.1002/mds.26708

[24] Louis ED, Clark LN, and Ottman R, “Familial versus sporadic essential tremor: What patterns can one decipher in age of onset?” Neuroepidemiology, vol. 44, no. 3, pp. 166–172, 2015. 25967236 10.1159/000381807 PMC4764084

[25] Alty J, Cosgrove J, Thorpe D, and Kempster P, “How to use pen and paper tasks to aid tremor diagnosis in the clinic,” Practical Neurol, vol. 17, no. 6, pp. 456–463, 12. 2017. 10.1136/practneurol-2017-001719 PMC5739823 28844041

[26] Michalec M, Hernandez N, Clark LN, and Louis ED, “The spiral axis as a clinical tool to distinguish essential tremor from dystonia cases,” Parkinsonism Rel. Disorders, vol. 20, no. 5, pp. 541–544, 5 2014. 10.1016/j.parkreldis.2014.01.021 PMC4028420 24560600

[27] Cerasa A and Quattrone A, “Linking essential tremor to the cerebellum—Neuroimaging evidence,” Cerebellum, vol. 15, no. 3, pp. 263–275, 6. 2016. 26626626 10.1007/s12311-015-0739-8

[28] Schmouth J-F, Dion PA, and Rouleau GA, “Genetics of essential tremor: From phenotype to genes, insights from both human and mouse studies,” Prog. Neurobiol, vols. 119-120, pp. 1–19, 8. 2014. 10.1016/j.pneurobio.2014.05.001 24820404

[29] Hallett M, “Tremor: Pathophysiology,” Parkinsonism Rel. Disorders, vol. 20, pp. S118–S122, 1. 2014. 10.1016/S1353-8020(13)70029-4 24262161

[30] Helmich RC, Toni I, Deuschl G, and Bloem BR, “The pathophysiology of essential tremor and Parkinson’s tremor,” Current Neurol. Neurosci. Rep, vol. 13, no. 9, p. 378, 7. 2013. 10.1007/s11910-013-0378-8 23893097

[31] Brown AM, White JJ, van der Heijden ME, Zhou J, Lin T, and Sillitoe RV, “Purkinje cell misfiring generates high-amplitude action tremors that are corrected by cerebellar deep brain stimulation,” eLife, vol. 9, p. e51928, 3. 2020. 32180549 10.7554/eLife.51928 PMC7077982

[32] Pedrosa DJ, Brown P, Cagnan H, Visser-Vandewalle V, Wirths J, Timmermann L, and Brittain J-S, “A functional micro-electrode mapping of ventral thalamus in essential tremor,” Brain, vol. 141, no. 9, pp. 2644–2654, 9. 2018. 30052807 10.1093/brain/awy192 PMC6113647

[33] Puttaraksa G, Muceli S, Gallego JÁ, Holobar A, Charles SK, Pons JL, and Farina D, “Voluntary and tremorogenic inputs to motor neuron pools of agonist/antagonist muscles in essential tremor patients,” J. Neurophysiol, vol. 122, no. 5, pp. 2043–2053, 11. 2019. 31509467 10.1152/jn.00407.2019 PMC6998026

[34] Deuschl G, Bain P, and Brin M, “Consensus statement of the movement disorder society on tremor,” Movement Disorders, vol. 13, no. S3, pp. 2–23, 10. 2008. 10.1002/mds.870131303 9827589

[35] Zhang D, Poignet P, Bo AP, and Ang WT, “Exploring peripheral mechanism of tremor on neuromusculoskeletal model: A general simulation study,” IEEE Trans. Biomed. Eng, vol. 56, no. 10, pp. 2359–2369, 10. 2009. 19535320 10.1109/TBME.2009.2023979

[36] Corie TH and Charles SK, “Simulated tremor propagation in the upper limb: From muscle activity to joint displacement,” J. Biomech. Eng, vol. 141, no. 8, pp. 0810011–08100117, 8. 2019. 10.1115/1.4043442 PMC6528735 30964940

[37] Pigg AC, Thompson-Westra J, Mente K, Maurer CW, Haubenberger D, Hallett M, and Charles SK, “Distribution of tremor among the major degrees of freedom of the upper limb in subjects with essential tremor,” Clin. Neurophysiol, vol. 131, no. 11, pp. 2700–2712, 11. 2020. 33010725 10.1016/j.clinph.2020.08.010 PMC7606740

[38] Sharma S and Pandey S, “Treatment of essential tremor: Current status,” Postgraduate Med. J, vol. 96, no. 1132, p. 84, 2020. 10.1136/postgradmedj-2019-136647 31575730

[39] Dallapiazza RF, Lee DJ, De Vloo P, Fomenko A, Hamani C, Hodaie M, Kalia SK, Fasano A, and Lozano AM, “Outcomes from stereotactic surgery for essential tremor,” J. Neurol., Neurosurg. Psychiatry, vol. 90, no. 4, p. 474, 2019. 30337440 10.1136/jnnp-2018-318240 PMC6581115

[40] Flora ED, Perera CL, Cameron AL, and Maddern GJ, “Deep brain stimulation for essential tremor: A systematic review,” Movement Disorders, vol. 25, no. 11, pp. 1550–1559, 8. 2010. 20623768 10.1002/mds.23195

[41] Barbe MT, Reker P, Hamacher S, Franklin J, Kraus D, Dembek TA, Becker J, Steffen JK, Allert N, Wirths J, Dafsari HS, Voges J, Fink GR, Visser-Vandewalle V, and Timmermann L, “DBS of the PSA and the VIM in essential tremor: A randomized, double-blind, crossover trial,” Neurology, vol. 91, no. 6, pp. e543–e550, 8. 2018. 29970404 10.1212/WNL.0000000000005956

[42] Kestenbaum M, Ford B, and Louis ED, “Estimating the proportion of essential tremor and Parkinson’s disease patients undergoing deep brain stimulation surgery: Five-year data from Columbia university medical center (2009–2014),” Movement Disorders Clin. Pract, vol. 2, no. 4, pp. 384–387, 12. 2015. 10.1002/mdc3.12185 PMC5571873 28845438

[43] Miterko LN , “Consensus paper: Experimental neurostimulation of the cerebellum,” Cerebellum, vol. 18, no. 6, pp. 1064–1097, 12. 2019. 31165428 10.1007/s12311-019-01041-5 PMC6867990

[44] Grimaldi G and Manto M, “Neurological tremor: Sensors, signal processing and emerging applications,” Sensors, vol. 10, no. 2, pp. 1399–1422, 2. 2010. 22205874 10.3390/s100201399 PMC3244020

[45] Basu I, Tuninetti D, Graupe D, and Slavin KV, “Adaptive control of deep brain stimulator for essential tremor: Entropy-based tremor prediction using surface-EMG,” in Proc. Annu. Int. Conf IEEE Eng. Med. Biol. Soc, 8. 2011, pp. 7711–7714. 10.1109/IEMBS.2011.6091900 22256125

[46] Basu I, Graupe D, Tuninetti D, Shukla P, Slavin KV, Metman LV, and Corcos DM, “Pathological tremor prediction using surface electromyogram and acceleration: Potential use in ‘ON-OFF’ demand driven deep brain stimulator design,” J. Neural Eng, vol. 10, no. 3, 6. 2013, Art. no. 036019. 10.1088/1741-2560/10/3/036019 PMC4524567 23658233

[47] Maneski LP, Jorgovanovic N, Ilic V, Došen S, Keller T, Popovic MB, and Popovic DB, “Electrical stimulation for the suppression of pathological tremor,” Med. Biol. Eng. Comput, vol. 49, no. 10, 7. 2011, Art. no. 1187. 10.1007/s11517-011-0803-6 21755318

[48] Zhang D, Poignet P, Widjaja F, and Ang WT, “Neural oscillator based control for pathological tremor suppression via functional electrical stimulation,” Control Eng. Pract, vol. 19, no. 1, pp. 74–88, 1. 2011.

[49] Dideriksen JL, Gianfelici F, Maneski LZP, and Farina D, “EMG-based characterization of pathological tremor using the iterated Hilbert transform,” IEEE Trans. Biomed. Eng, vol. 58, no. 10, pp. 2911–2921, 10. 2011. 21803679 10.1109/TBME.2011.2163069

[50] Gallego JA, Rocon E, Ibanez J, Dideriksen JL, Koutsou AD, Paradiso R, Popovic MB, Belda-Lois JM, Gianfelici F, Farina D, Popovic DB, Manto M, D’Alessio T, and Pons JL, “A soft wearable robot for tremor assessment and suppression,”. in Proc. IEEE Int. Conf. Robot. Automat, 5 2011, pp. 2249–2254.

[51] Elble RJ and McNames J, “Using portable transducers to measure tremor severity,” Tremor Other Hyperkinetic Movements, vol. 6, p. 375, 5 2016. 27257514 10.7916/D8DR2VCC PMC4872171

[52] Elble RJ, “Gravitational artifact in accelerometric measurements of tremor,” Clin. Neurophysiol, vol. 116, no. 7, pp. 1638–1643, 7. 2005. 15905122 10.1016/j.clinph.2005.03.014

[53] Isaacson SH , “Prospective home-use study on non-invasive neuromodulation therapy for essential tremor,” Tremor Other Hyperkinetic Movements, vol. 10, p. 29, 8. 2020. 32864188 10.5334/tohm.59 PMC7427656

[54] Gallego J, Rocon E, Roa JO, Moreno J, and Pons JL, “Real-time estimation of pathological tremor parameters from gyroscope data,” Sensors, vol. 10, no. 3, pp. 2129–2149, 3. 2010. 22294919 10.3390/s100302129 PMC3264472

[55] McGurrin P, McNames J, Wu T, Hallett M, and Haubenberger D, “Quantifying tremor in essential tremor using inertial sensors—Validation of an algorithm,” IEEE J. Transl. Eng. Health Med, vol. 9, 2021, Art. no. 2700110. 10.1109/JTEHM.2020.3032924 PMC7608862 33150096

[56] López-Blanco R, Velasco MA, Méndez-Guerrero A, Romero JP, del Castillo MD, Serrano JI, Benito-León J, Bermejo-Pareja F, and Rocon E, “Essential tremor quantification based on the combined use of a smartphone and a smartwatch: The NetMD study,” J. Neurosci. Methods, vol. 303, pp. 95–102, 6. 2018. 29481820 10.1016/j.jneumeth.2018.02.015

[57] Popović LZ, šekara TB, and Popović MB, “Adaptive band-pass filter (ABPF) for tremor extraction from inertial sensor data,” Comput. Methods Programs Biomed, vol. 99, no. 3, pp. 298–305, 9. 2010. 20430466 10.1016/j.cmpb.2010.03.018

[58] Du J, Gerdtman C, and Lindén M, “Signal quality improvement algorithms for MEMS gyroscope-based human motion analysis systems: A systematic review,” Sensors, vol. 18, no. 4, p. 1123, 4. 2018. 10.3390/s18041123 PMC5948938 29642412

[59] Fromme NP, Camenzind M, Riener R, and Rossi RM, “Need for mechanically and ergonomically enhanced tremor-suppression orthoses for the upper limb: A systematic review,” J. Neuroeng. Rehabil, vol. 16, no. 1, p. 93, 7. 2019. 31319893 10.1186/s12984-019-0543-7 PMC6639950

[60] Abbasi M, Afsharfard A, Arasteh R, and Safaie J, “Design of a noninvasive and smart hand tremor attenuation system with active control: A simulation study,” Med. Biol. Eng. Comput, vol. 56, no. 7, pp. 1315–1324, 7. 2018. 29297138 10.1007/s11517-017-1769-9

[61] Gironell A, Kulisevsky J, Lorenzo J, Barbanoj M, Pascual-Sedano B, and Otermin P, “Transcranial magnetic stimulation of the cerebellum in essential tremor: A controlled study,” Arch. Neurol, vol. 59, no. 3, pp. 413–417, 3. 2002. 11890845 10.1001/archneur.59.3.413

[62] Olfati N, Shoeibi A, Abdollahian E, Ahmadi H, Hoseini A, Akhlaghi S, Vakili V, Foroughipour M, Rezaeitalab F, Farzadfard M-T, Layegh P, and Naseri S, “Cerebellar repetitive transcranial magnetic stimulation (rTMS) for essential tremor: A double-blind, sham-controlled, crossover, add-on clinical trial,” Brain Stimulation, vol. 13, no. 1, pp. 190–196, 1. 2020. 31624048 10.1016/j.brs.2019.10.003

[63] Yilmaz NH, Polat B, and Hanoglu L, “Transcranial direct current stimulation in the treatment of essential tremor: An open-label study,” Neurologist, vol. 21, no. 2, pp. 28–29, 3. 2016. 26926852 10.1097/NRL.0000000000000070

[64] Chalah MA, Lefaucheur J-P, and Ayache SS, “Non-invasive central and peripheral stimulation: New hope for essential tremor?” Frontiers Neurosci., vol. 9, p. 440, 11. 2015. 10.3389/fnins.2015.00440 PMC4649015 26635516

[65] Prochazka A, Elek J, and Javidan M, “Attenuation of pathological tremors by functional electrical stimulation I: Method,” Ann. Biomed. Eng, vol. 20, no. 2, pp. 205–224, 3. 1992. 1575377 10.1007/BF02368521

[66] Javidan M, Elek J, and Prochazka A, “Attenuation of pathological tremors by functional electrical stimulation II: Clinical evaluation,” Ann. Biomed. Eng, vol. 20, no. 2, pp. 225–236, 3. 1992. 1575378 10.1007/BF02368522

[67] Britton TC, Thompson PD, Day BL, Rothwell JC, Findley LJ, and Marsden CD, “Modulation of postural tremors at the wrist by supramaximal electrical median nerve shocks in essential tremor, Parkinson’s disease and normal subjects mimicking tremor,” J. Neurol. Neurosurg. Psychiatry, vol. 56, no. 10, p. 1085, 1993. 8410007 10.1136/jnnp.56.10.1085 PMC1015237

[68] Dosen S, Muceli S, Dideriksen JL, Romero JP, Rocon E, Pons J, and Farina D, “Online tremor suppression using electromyography and low-level electrical stimulation,” IEEE Trans. Neural Syst. Rehabil. Eng, vol. 23, no. 3, pp. 385–395, 5 2015. 25051555 10.1109/TNSRE.2014.2328296

[69] Widjaja F, Shee CY, Ang WT, Au WL, and Poignet P, “Sensing of pathological tremor using surface electromyography and accelerometer for real-time attenuation,” J. Mech. Med. Biol, vol. 11, no. 5, pp. 1347–1371, 12. 2011.

[70] Zhang D and Ang W, “Tremor suppression of elbow joint via functional electrical stimulation: A simulation study,” in Proc. IEEEInt. Conf. Automat. Sci. Eng, 10. 2006, pp. 182–187.

[71] Copur EH, Freeman CT, Chu B, and Laila DS, “Repetitive control of electrical stimulation for tremor suppression,” IEEE Trans. Control Syst. Technol, vol. 27, no. 2, pp. 540–552, 3. 2019.

[72] Brunetti F, Garay A, Moreno JC, and Pons JL, “Enhancing functional electrical stimulation for emerging rehabilitation robotics in the framework of hyper project,” in Proc. IEEE Int. Conf. Rehabil. Robot, 6. 2011, pp. 1–6. 10.1109/ICORR.2011.5975370 22275574

[73] Holmgren C, Carlsson T, Mannheimer C, and Edvardsson N, “Risk of interference from transcutaneous electrical nerve stimulation on the sensing function of implantable defibrillators,” Pacing Clin. Electrophysiol, vol. 31, no. 2, pp. 151–158, 2. 2008. 18233966 10.1111/j.1540-8159.2007.00962.x

[74] Badger J, Taylor P, and Swain I, “The safety of electrical stimulation in patients with pacemakers and implantable cardioverter defibrillators: A systematic review,” J. Rehabil. Assistive Technol. Eng, vol. 4, pp. 1–9, 12. 2017. 10.1177/2055668317745498 PMC6453072 31186945

[75] Egger F, Hofer C, Hammerle FP, Löfler S, Nürnberg M, Fiedler L, Kriz R, Kern H, and Huber K, “Influence of electrical stimulation therapy on permanent pacemaker function,” Wiener klinische Wochenschrift, vol. 131, nos. 13–14, pp. 313–320, 7. 2019. 31025164 10.1007/s00508-019-1494-5

[76] Taylor PN, Burridge JH, Dunkerley AL, Lamb A, Wood DE, Norton JA, and Swain ID, “Patients’ perceptions of the odstock dropped foot stimulator (ODFS),” Clin. Rehabil, vol. 13, no. 5, pp. 439–446, 10. 1999. 10498351 10.1191/026921599677086409

[77] Lambrecht S, Gallego JA, Rocon E, and Pons JL, “Automatic real-time monitoring and assessment of tremor parameters in the upper limb from orientation data,” Frontiers Neurosci, vol. 8, p. 221, 7. 2014. 10.3389/fnins.2014.00221 PMC4110507 25120424

[78] (2019). Health Care Professional FAQs. [Online]. Available: https://calatrio.com/healthcare-professionals/frequently-asked-questions/

